# Spontaneous lymphoblastoid cell lines from patients with Epstein-Barr virus infection show highly variable proliferation characteristics that correlate with the expression levels of viral microRNAs

**DOI:** 10.1371/journal.pone.0222847

**Published:** 2019-09-30

**Authors:** Susanne Delecluse, Jiyang Yu, Katharina Bernhardt, Janina Haar, Remy Poirey, Ming-Han Tsai, Rama Kiblawi, Annette Kopp-Schneider, Paul Schnitzler, Martin Zeier, Peter Dreger, Patrick Wuchter, Olcay Cem Bulut, Uta Behrends, Henri-Jacques Delecluse

**Affiliations:** 1 German Cancer Research Centre (DKFZ) Unit F100, Heidelberg, Germany; 2 Institut National de la Santé et de la Recherche Médicale (INSERM) Unit U1074, Heidelberg, Germany; 3 German Centre for Infection Research (DZIF), Braunschweig, Germany; 4 Nierenzentrum Heidelberg, Heidelberg, Germany; 5 German Cancer Research Centre (DKFZ), Unit C060, Heidelberg, Germany; 6 Center for Infectious Diseases, Virology, University Hospital Heidelberg, Heidelberg, Germany; 7 Department of Medicine V, University of Heidelberg, Heidelberg, Germany; 8 Institute of Transfusion Medicine and Immunology, German Red Cross Blood Donor Service Baden-Württemberg–Hessen, Medical Faculty Mannheim, Heidelberg University, Germany; 9 Department of Otorhinolaryngology, Head and Neck Surgery, University Hospital Heidelberg, Heidelberg, Germany; 10 Children’s Hospital Klinikum Rechts der Isar, Technische Universitaet Muenchen, Munich, Germany; University of Nebraska-Lincoln, UNITED STATES

## Abstract

The Epstein-Barr virus (EBV) induces B-cell proliferation with high efficiency through expression of latent proteins and microRNAs. This process takes place *in vivo* soon after infection, presumably to expand the virus reservoir, but can also induce pathologies, e.g. an infectious mononucleosis (IM) syndrome after primary infection or a B-cell lymphoproliferation in immunosuppressed individuals. In this paper, we investigated the growth characteristics of EBV-infected B-cells isolated from transplant recipients or patients with IM. We found that these cells grew and withstood apoptosis at highly variable rates, suggesting that the expansion rate of the infected B-cells widely varies between individuals, thereby influencing the size of the B-cell reservoir and the ability to form tumors in infected individuals. All viruses investigated were type 1 and genetically close to western strains. EBV-infected B-cells expressed the transforming EBV latent genes and microRNAs (miRNAs) at variable levels. We found that the B-cell growth rates positively correlated with the BHRF1 miRNA levels. Comparative studies showed that infected B-cells derived from transplant recipients with iEBVL on average expressed higher levels of EBV miR-BHRF1 miRNAs and grew more rapidly than B-cells from IM patients, suggesting infection by more transforming viruses. Altogether, these findings suggest that EBV infection has a highly variable impact on the B-cell compartment that probably reflects the genetic diversity of both the virus and the host. It also demonstrates the unexpected finding that B-cells from different individuals can grow at different speed under the influence of the same virus infection.

## Introduction

The Epstein-Barr virus (EBV) infects the large majority of the human population [1]. A primary EBV infection is usually clinically silent, except if delayed until adolescence. In that case, a generally self-limiting infectious mononucleosis (IM) syndrome can develop [2]. Upon infection, the virus targets primary B-cells in which it induces an unlimited B-cell proliferation [3]. This process depends on the expression of the

Epstein-Barr nuclear antigens (EBNA) and of the latent membrane proteins (LMP) subfamilies [3]. EBV MicroRNAs (miRNAs) have also been found to modulate the transforming potential of the virus [4, 5]. Whilst the miRNAs clustered around the BHRF1 gene potentiate B-cell proliferation, those clustered in the BART transcripts have the opposite effect, probably through their action on LMP1 [6–8].

An EBV infection rarely causes pathologies in individuals with a healthy immune system because infected cells are destroyed by the immune response [1]. Although it curbs B-cell proliferation, this response cannot eradicate the virus from the host in which it persists in memory B-cells, and perhaps in other cell types to form the virus reservoir [9]. In immunosuppressed patients, the balance between EBV and its host is shifted towards the virus and infected B-cells can resume proliferation [10], resulting in an increase in the number of infected B-cells in the peripheral blood and an increased EBV load (iEBVL) in this compartment [11, 12]. In a smaller fraction of patients, it can also give rise to the development of different types of EBV-positive lymphoproliferative diseases [13, 14]. Thus, the EBV-induced B-cell proliferation is at the heart of the interactions between the virus and its host in both physiology and disease. However, very little is known about the growth characteristics of EBV-transformed B-cells in infected individuals. In this paper, we report the *in vitro* growth characteristics of a panel of 25 spontaneously proliferating EBV-infected B-cells (sLCLs) isolated from the blood of 13 transplanted patients with an increased EBV load or PTLD and from 9 patients undergoing IM. We found that different cell samples display remarkably variable growth characteristics that reflect a great heterogeneity of viral protein and miRNA expression.

## Material and methods

### Establishment of cell lines

Fourteen independent spontaneously growing EBV-positive B-cell lines (sLCLs) were established from the peripheral blood of thirteen immunosuppressed transplanted patients with an increased EBV load (> 1000 EBV genome copies/ml) (sLCL1 to -14) (Table 1), ten from nine immunocompetent patients suffering from IM (IM-1 to -10) and one from a patient with PTLD. Patients suffering from IM were diagnosed according to clinical symptoms together with the detection of EBV specific IgM and IgG antibodies. B-cells were positively selected with a CD19-specific magnetic bead system as previously described [15]. 1x10^5^ B-cells were seeded onto normal human dermal fibroblasts feeder cells (Promo cell) in 96 well plates as earlier attempts without feeder cells proved unsuccessful. Control cell lines included LCLs generated from a single individual with the laboratory EBV strains M81 or B95-8, and the Burkitt’s lymphoma cell lines Raji, Oku (both EBV-positive) and Elijah (EBV-negative) [16–18]. Cell lines were cultured in RPMI-1640 medium (Gibco) supplemented with 10% fetal bovine serum (Sigma). The work has been carried out in accordance with the Declaration of Helsinki. All human subjects gave their written informed consent. The ethics committee of the University of Heidelberg and of the Klinikum rechts der Isar, Technische Universitaet Muenchen, approved the study (approvals S/005-2014 and 112/14).

**Table 1:** Characteristics of the studied patients (sLCL-1 to-14) with an iEBVL and one patient with a post-transplant lymphoproliferative disorder (PTLD-1).

Patient number	Age	Sex	Country of origin	Transplanted organ	Time from transplantation to iEBVL (in days)	EBV cop/ml WB	Immunosuppressive regimen	PTLD	status at last FU
sLCL-1	66	m	Germany	SCT	26	3150	CSA, MTX	-	alive
sLCL-2	66	m	Germany	SCT	26	3150	CSA, MTX	-	alive
sLCL-3	56	f	Germany	KT	7456	9870	CyA, ST	+^a^	alive
sLCL-4	66	f	Spain	KT	1821	18600	CSA, MMF, ST	-	alive
sLCL-5	64	m	Germany	SCT	29	1000	FK, MMF	-	dead (relapsed ALL)
sLCL-6	66	f	Germany	KT	3024	1730	ST	-	alive
sLCL-7	68	m	Germany	SCT	26	2390	CSA	-	alive
sLCL-8	46	f	Russia	SCT	41	2330	CSA	-	alive
sLCL-9	64	m	Germany	SCT	35	2600	CSA	-	alive
sLCL-10	60	f	Germany	KT	2853	1920	BELA/ST	-	alive
sLCL-11	63	m	Germany	SCT	273	16200	FK	-	alive
sLCL-12	56	f	Turkey	KT	18	8450	CSA, MMF, ST	-	alive
sLCL-13	32	m	Germany	KT	22	22600	CSA, ST	-	alive
sLCL-14	37	m	Germany	KT	810	16400	FK, MMF, ST	-	alive
PTLD-1	49	m	Germany	SCT	27	10700	CSA	+	dead (PTLD)

iEBVL: increased EBV load, CSA: Cyclosporin A, MMF: Mycofenolate mofetil, FK: Tacrolimus, SIR: Sirolimus, AZA: Azathioprine, MTX: Methotrexate, BELA: Belatacept, KT: kidney transplantation, SCT: stem cell transplantation, ST: Steroid, m: male, f: female, cop: copies, WB: whole blood, FU: follow up, ALL: acute lymphoblastic leukemia.

^a^T-cell PTLD, not EBV-associated.

### TUNEL assay and Western blot

Apoptosis was induced by treating the cells for 24 hours with etoposide (4 microg/ml), ionomycin (5 microg/ml), or staurosporine (4 microg/ml). Cells were fixed with 4% paraformaldehyde for 1 hour at room temperature. TdT-mediated dUTP-X nick end labelling (TUNEL) was performed according to the manufacturer’s instructions using an In Situ Cell death Detection Kit (Roche, REF. 12156722910). Slides were embedded with 90% glycerol and analysed using a Leica epifluorescence microscope. The number of apoptotic cells was determined by manual counting and given as percentage of apoptotic cells. For correlation studies the results were log-transformed. Western blots for viral proteins were performed as described before [19]. For the detection of EBNA1, EBNA2, LMP1, and BHRF1, 50 μg proteins were treated with beta-mercaptoethanol and loaded onto a 10% or a 15% SDS acrylamide gel. Antibodies used are given in the Table A in S1 File. Antibodies against actin were used as loading controls. We quantified the protein signals using ImageJ software.

### FISH

For fluorescence *in situ* hybridization (FISH), cells were treated with an hypotonic solution containing 0.0075 m KCl, repeatedly fixed in ice cold methanol: acetic acid (vol:vol 75:25) and applied onto glass slides. We generated an EBV-specific probe by nick translation of the complete B 95–8 BAC as described before [20]. Denaturation of cells was performed in 2 × SSC 70% deionized formamide for 2 min at 75°C and followed by dehydration in an increasingly concentrated alcohol series. The probe was denatured at 75°C for 5 min, applied onto dehydrated cells, and incubated overnight at 37°C. Slides were then washed in 4 × SSC. The probe was detected by streptavidin-conjugated fluorescein Alexa 488 (Life Technologies, Carlsbad, California, USA). Slides were analyzed using a Leica epifluorescence microscope (Wetzlar, Germany).

### qPCR

DNA extracted from spontaneous cell lines was used for qPCR analyses to determine the EBV viral load. We performed TaqMan qPCR analyses using primers and probes designed to amplify the EBV polymerase (BALF5, forward primer 5’-CTTTGGCGCGGATCCTC-3’, reverse primer 5’-AGTCCTTCTTGGCTAGTCTGTTGAC-3’, probe 5’-Fam-CATCAAGAAGCTGCTGGCGGCC-Tamra-3) or the BGLF5 gene (forward primer 5’-CCTCTTTTCCAAGTCAGAATTGAC-3’, reverse primer 5’-TGACCTCTTGCATGGCCTCT-3’, probe 5’-Fam-CCATCTACCCATCCTACACTGCTTTACA-Tamra-3’). Amplification reactions were performed in a total volume of 25 μl, including 12.5 μl of TaqMan universal PCR master mix (Applied Biosystems, Waltham, Massachusetts, USA), 2.5 μl each of forward and reverse primers (2 μm), 1 μl of 5 μm FAM‐labeled Pol‐probe, 1.5 μl of water, and 5 μl of buffer containing the purified DNA samples. After initial activation of the DNA polymerase for 10 min at 95°C, samples were amplified for 40 cycles (15 s at 95°C and 60 s at 60°C). The ABI 7300 real-time PCR system (Applied Biosystems) was used for detection of the fluorescent signals. A serial dilution of an EBV bacterial artificial chromosome (BAC) preparation (p2089) was included to calculate the viral DNA content of the different supernatants.

### RNA extraction/reverse transcription

RNA extraction for cell lines was performed using Trizol reagent and processed as described before [21]. In brief 5 x 10^6^ cells were pelleted, washed in PBS and lysed using 1 ml Trizol. After addition of 200 μl chloroform, the samples were centrifugated for 15 min at 12000 rpm. The aqueous phase was collected and the RNA precipitated with isopropanol. Reverse transcription was performed using a TaqMan^R^ MicroRNA Reverse Transcription Kit (Thermo Fisher Scientific Inc, USA). Reverse stem-loop primers are given in the supplementary Table B in S1 File. A total of 110 ng RNA was used for reverse transcription.

### Stem-loop RT-qPCR

Multiple EBV miRNAs were amplified as described by Cosmopoulos et al. [22]. Sequences of forward and reverse primers as well as probes used are given in Table B in S1 File. The snoRNA RNU48 was amplified as an internal control to normalize for the amount of RNA present in each sample [6]. Reverse transcription and qPCR for RNU48 were performed as indicated by the manufacturer (TaqMan^R^ MicroRNA control Assay, Thermo Fisher Scientific Inc).

### Flow cytometry

Flow cytometry was used to characterize cell surface receptor expression on the sLCLs. Briefly, 1 x 10^6^ cells were washed in PBS. Cells were incubated on ice with a PE-coupled anti-CD19 antibody or an isotype control for 20 min and washed twice with PBS. After washing and filtering, cells were counted on a BD FACSCalibur flow cytometer (BD Biosciences). The BD CellQuest Pro software was used to analyse the cell populations (BD Biosciences).

### Sequencing and alignment

EBNA2-, EBNA3A-, BZLF1- and LMP1-specific sequences were cloned onto a pBlueScript II SK (+) vector (Adgene). The primer sequences used and the sequence positions in the reference EBV genome NC_007605.1 are given in the Table C in S1 File. The genome sequences of different EBV strains were downloaded from the NCBI database and aligned with the Multiple Alignment using Fast Fourier Transform (MAFFT) v7.419 software. The EBNA2-, EBNA3A-, BZLF1- and LMP1 sequences from each of these viral strains were aligned against each other with the MacVector software version 16 using ClustalW. A phylogenetic tree from these sequences was generated with the MEGA7 software using minimum-evolution (ME) method (Bootstrap 1000). The ME tree was searched using the close-neighbor-interchange (CNI) algorithm at a search level of 2.

### Statistics

Statistical analyses were performed using the GraphPad Prism 6 software. As indicated in the figure legends, some results are given as arithmetic mean with standard error (SEM). We performed correlation studies by calculating the Pearson’s correlation coefficient. For this analysis, both sets of values were log-transformed. We also compared the properties of sLCLs established from the blood of transplant recipients with iEBVL and from the blood of IM patients using a non-paired t-test.

## Results

### 1) Growth characteristics of a panel of EBV-positive B-cell lines established from transplant recipients and from patients with IM

**Fig 1:**
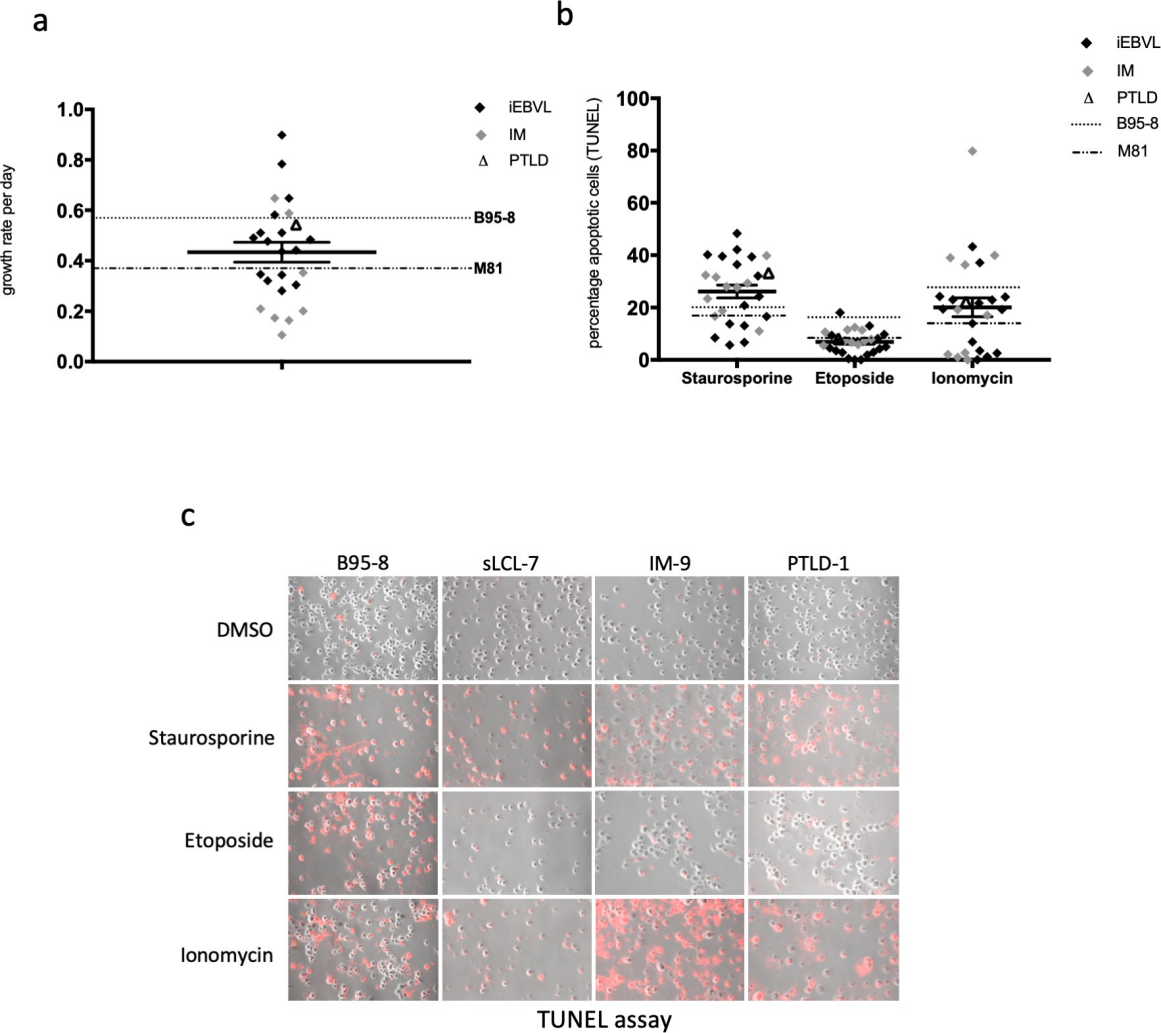
The cell growth rate and resistance to apoptosis vary widely across a panel of EBV-transformed B-cells isolated from patients with an increased EBV load after transplantation or from IM patients. (a) We seeded infected B-cells from transplanted patients with increased EBV load (iEBVL) or PTLD and from IM patients under optimal growth conditions and calculated their growth rate per day. We monitored in parallel the growth of B-cells from a blood sample infected with either B95-8 or M81, two viruses known to induce rapid and slow cell growth, respectively. The dot plot shows the growth rate per day of each sample. We also indicate the mean of these values and their standard error. (b) Apoptosis was induced in the same panel of B-cells using three different drugs. The percentage of apoptotic cells in each case was determined by a TUNEL assay. The results of the assays are summarized in three dot plots. (c) Examples of stains with the TUNEL assay are shown.

We expanded EBV-infected B-cells from thirteen transplant recipients with an increased EBV load, whose clinical characteristics are given in Table 1. These B-cells generated fourteen independent sLCLs. To this end, we seeded purified B-cells from these patients in cluster plates coated with feeder cells. As assessed by the expression of the CD19 B-cell marker, we obtained multiple proliferating B-cell lines within one to two weeks (Fig A in S1 File). We investigated the growth characteristics of these cell lines under optimal cell concentration (3x10^5^/ml) and medium supply, together with a panel of ten sLCLs established from nine individuals with IM and one line from a patient with PTLD. We also included two LCLs generated by infection of B-cells from a healthy donor with B95-8 or M81, two laboratory EBV strains that display different growth characteristics [23]. We found that this panel of sLCLs differed by more than eightfold in their growth rates (Fig 1A). The growth of many of these cell lines was intermediate between the LCL generated with B95-8 and M81, the former being more efficient at inducing cell growth than the second. We then studied the ability of the sLCLs to withstand a proapoptotic stimulus by treating them with etoposide, ionomycin or staurosporine, three drugs that induce apoptosis through activation of different pathways (Fig 1B) [24–26]. One day after treatment, cells were subjected to a TUNEL assay to identify apoptotic cells (Fig 1C). Here again, the panel of sLCLs was very heterogeneous with a percentage of apoptotic cells ranging from 0.01 to 43.3% after ionomycin treatment (Fig 1B).

### 2) The panel of sLCLs consists of type I viruses close to European and North American isolates

**Fig 2:**
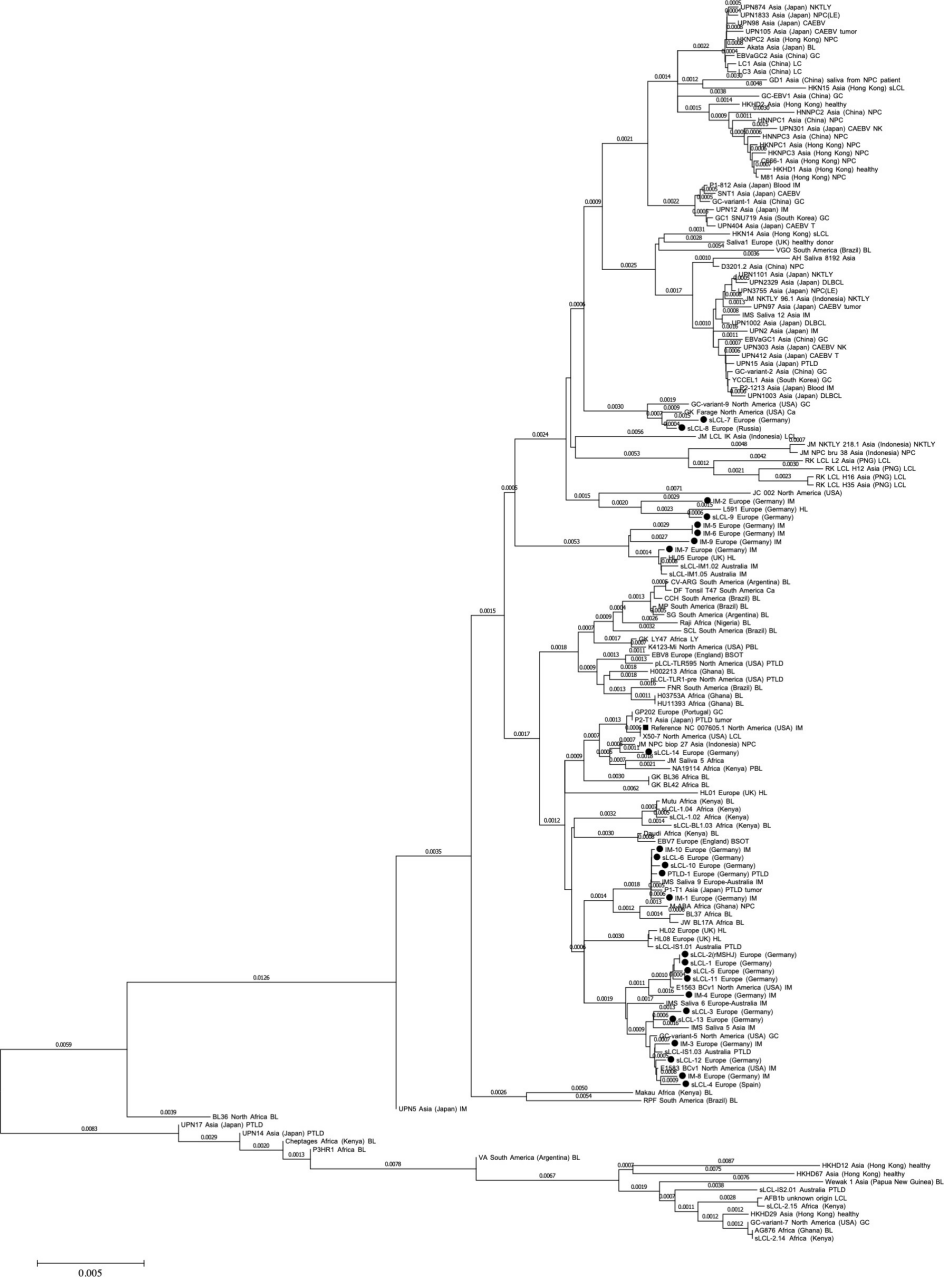
Genetic classification of spontaneous LCLs from patients with IM and iEBVL. The genetic tree shows the genetic distance between our panel of spontaneous LCLs (black dots) and published EBV strains after comparison of the sequences from four of their genes (EBNA2, EBNA3, BZLF1, LMP1).

Type 1 EBV viruses are more transforming than type 2 [27]. Moreover, we previously showed that B95-8 isolated from a US patient is more transforming than M81 that was isolated from a Chinese individual with nasopharyngeal carcinoma [23]. It was therefore important to genotype and classify our virus panel within the group of available EBV sequences [28]. To this end, we sequenced the four most polymorphic EBV genes i.e. EBNA2, EBNA3A, LMP1 and BZLF1. These sequences identify all viruses in the panel as type 1 viruses (Table 1). Moreover, we aligned the sequences of the four tested viral genes from our virus panel with those available in the literature and generated a genetic tree that shows the degree of genetic relation between these strains after deletion of their repeats. We first compared the tree generated with the sequence information from published EBV strains restricted to EBNA2, EBNA3A, LMP1 and BHRF1 with the tree obtained with their complete viral genomes. We found that the global arrangement of the different strains in the divergence tree is globally, if not perfectly, conserved between both methods, although the genetic distance between the strains varied in the two different approaches (Compare Fig 2 with Fig B in S1 File). Having validated the approach restricted to the EBNA2, EBNA3A, LMP1 and BZLF1 genes, we applied it to our panel of viruses. This confirmed that these strains are close to European and North American isolates (Fig 2).

### 3) Expression pattern of latent proteins and microRNAs in a panel of sLCLs

**Fig 3:**
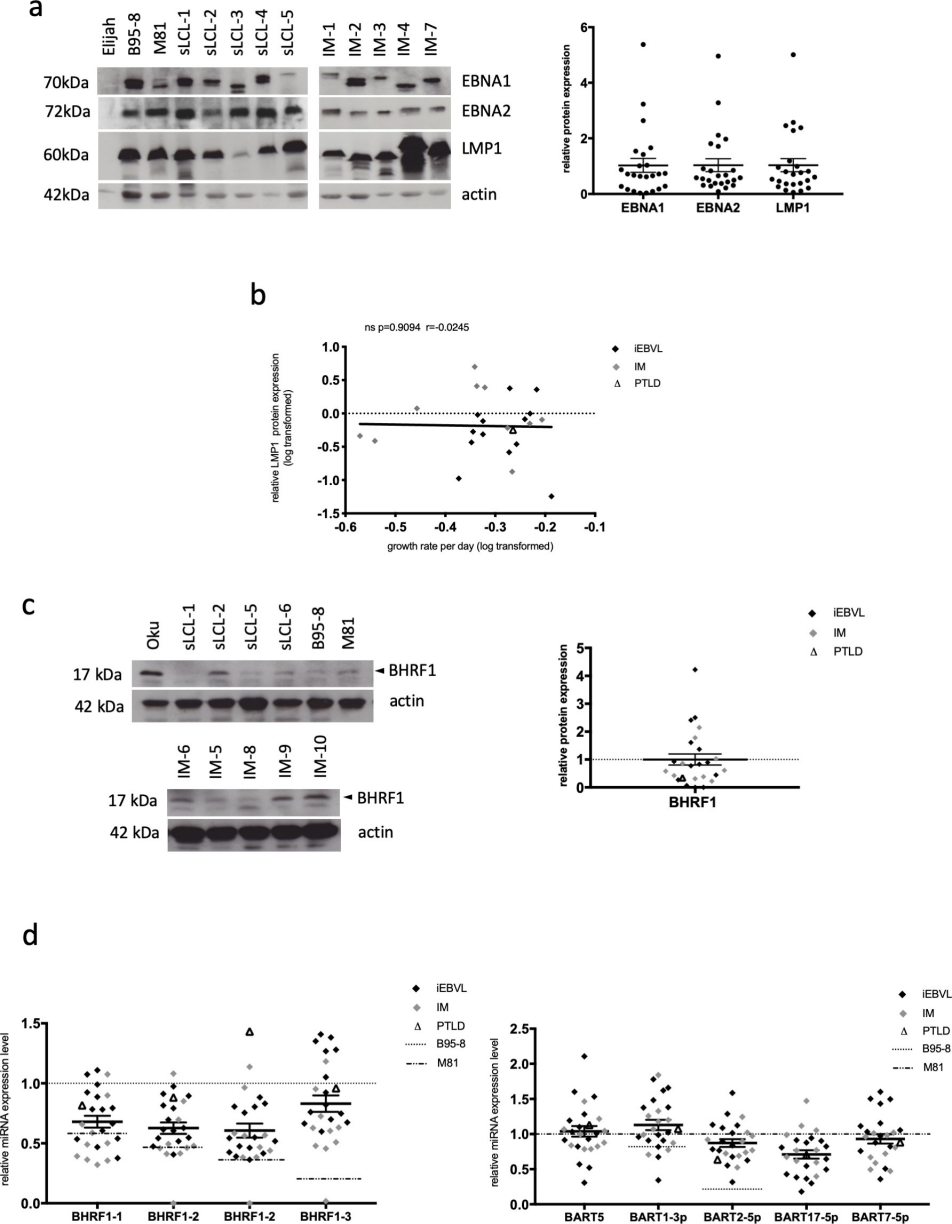
Latent protein and viral miRNA expression in spontaneous LCLs from patients with IM and iEBVL. (a) The figure shows the results of a western blot analysis that was performed on the panel of sLCLs with antibodies specific for EBNA1, EBNA2 and LMP1. Antibodies specific to actin were used as a loading control. Elijah served as an EBV-negative control. We quantified the protein signals using the ImageJ software. Signals were first given normalized to actin and then to the mean of the obtained values. Mean and standard error of protein expression levels are given in a dot plot. We show as an example the expression pattern in a subset of sLCLs from 10 IM (IM-1 to IM-4 and IM-7) and transplant recipients (sLCL-1 to sLCL-5), as well as in B-cells from a single individual infected with B95-8 or M81. (b) The graph shows the correlation coefficient between the growth rate of the investigated sLCLs and LMP1 protein expression levels. The values of the growth rates have been log-transformed to obtain a normal distribution. (c) Western blot analyses and protein signal quantification were performed for the BHRF1 protein. Signals were first given normalized to actin and then to the mean of the obtained values. Mean and standard error of protein expression levels are given as dot plot. The picture shows the results of the analysis on a subset of cell lines. The Oku cell line served as positive control as it is known to express high levels of this protein. (d) We measured the expression level of the four members of the BHRF1 miRNA cluster and of five members of the BART miRNA clusters using stem-loop RT-qPCR in all cell lines from patients with either iEBVL or IM. We also included the LCLs infected with M81 or B95-8 as controls. The dot plots summarize the expression levels relative to those observed in cells infected with B95-8 for the BHRF1 miRNA expression and relative to M81-infected cells for the BART miRNAs. We also indicated the average miRNA expression levels and standard error of the mean.

We then quantified the expression of EBV latent proteins, of BHRF1, an antiapoptotic viral protein, and of the EBV miRNAs by immunoblot and qPCR, respectively. As expected, iEBVL and IM cell lines all expressed EBNA1, EBNA2 and LMP1 that are characteristic for the latency III growth program (Fig 3A). Consistent with previously published reports, the molecular weights of EBNA2 and in particular of EBNA1 varied between the samples [29]. The range of LMP1 expression levels, a strongly transforming EBV protein, was markedly broad, with some cell lines expressing 40 times more LMP1 than others (IM-4 compared to sLCL-3). However, we could not find any correlation between LMP1 expression and the cellular growth rate (Fig 3B). Similar remarks apply to the BHRF1 protein (Fig 3C). In two patients, the BHRF1 protein could not be detected, in the others the BHRF1 expression varied up to fiftyfold. We quantified the expression of all four BHRF1 miRNAs and found that they were expressed in all sLCLs, except in IM-10 that lacked miR-BHRF1-2, -2* and -3 expression (Fig 3D, Fig C in S1 File). Thus, IM-10 expresses the BHRF1 protein but not the BHRF1 miRNAs. This is possible because the BHRF1 open reading frame and the BHRF1 miRNAs are located at different positions on the BHRF1 transcript. Sequencing confirmed the deletion of the BHRF1 microRNAs with preservation of the open reading frame. The expression of the BHRF1 miRNAs across the investigated B-cell samples extended over a 2.4 to 3.5-fold range, with a maximum for miR-BHRF1-1. This effect was even more pronounced for miR-BART1-3p, 2-5p, 5, 17-5p and -7, a set of miRNAs that are representative of each BART miRNA cluster (4.4 to 8.2-fold) (Fig 3D, Fig C in S1 File). As was true for the cell growth, the BHRF1 miRNA expression level was much higher in cells infected with B95-8 than in cells infected with M81 and most, but not all investigated samples displayed values that were intermediate between the values found in LCLs generated with these two laboratory strains. However, seven patient sLCLs expressed miR-BHRF1-3 at a higher level than the B95-8 LCL (Fig 3D). Reciprocally, miR-BART1-3p and -2-5p were expressed at higher levels in cells infected with M81 than in those infected with B95-8. Cells infected with B95-8 did not express the remaining miR-BARTs (5, 17-5p, and 7-5p) owing to a well-characterized deletion in the genome of this virus.

### 4) Correlation between EBV BHRF1 miRNA expression pattern and growth rate

**Fig 4:**
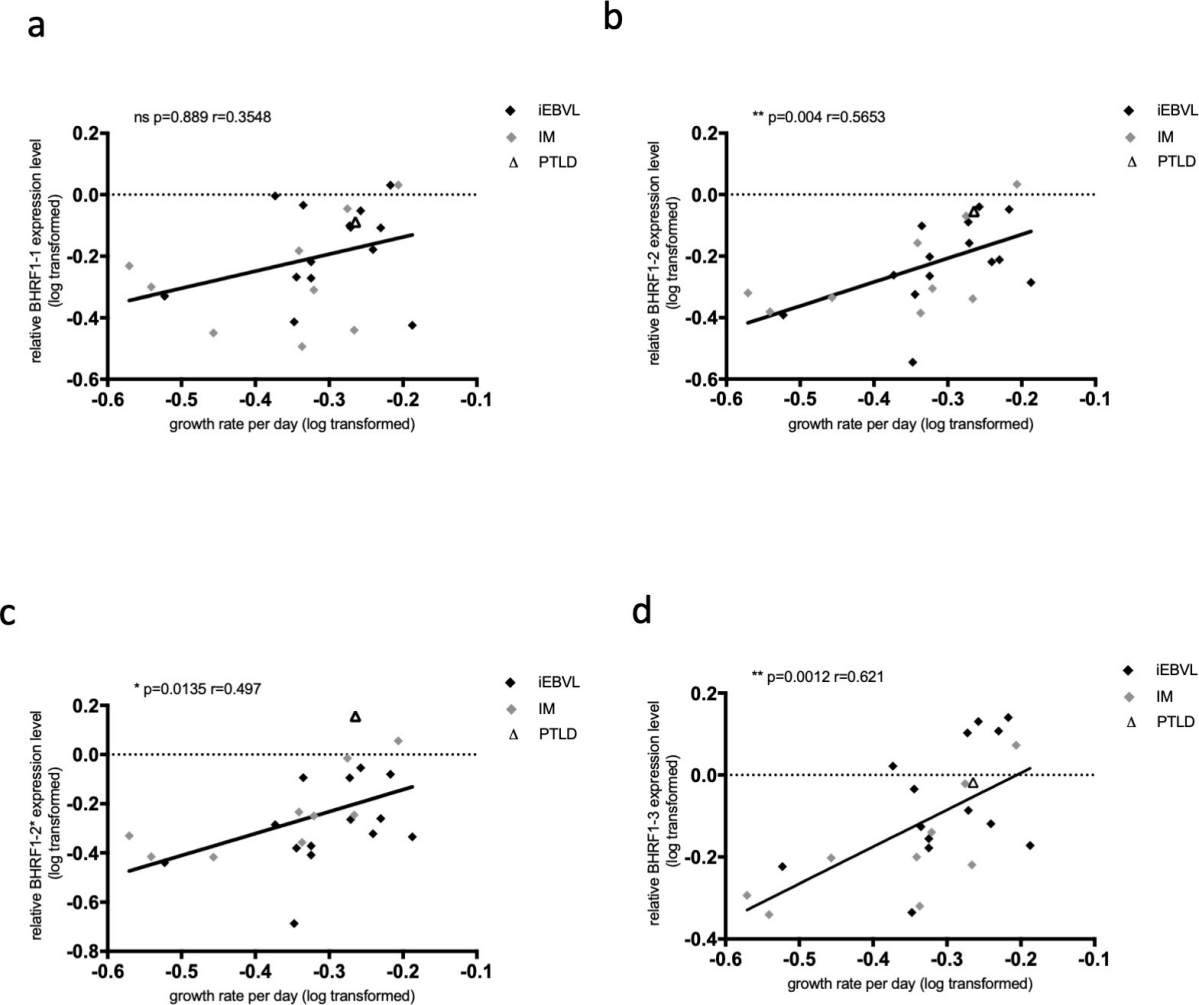
The growth of cells infected with EBV correlates with the BHRF1 miRNA expression level. We calculated the correlation coefficients between the growth rate of the investigated sLCLs and the expression of each of the BHRF1 miRNAs (a-d). The values of the growth rates have been log-transformed to obtain a normal distribution.

We then attempted to correlate the growth rate or the apoptosis rates of the sLCLs with the latent protein or miRNA expression levels. These analyses revealed a statistically significant positive correlation between the expression level of miR-BHRF1-2, miR-BHRF1-2* and miR-BHRF1-3 and the growth rate of the sLCLs with both parameters tested at the same time point (Fig 4B–4D). There was also a positive correlation with miR-BHRF1-1 but it did not reach statistical significance (Fig 4A). We could not find evidence of a correlation between the expression levels of the BHRF1 miRNAs and the apoptotic rates (Fig D in S1 File). Correlation studies between latent protein expression (EBNAs, LMP1, BHRF1 protein) and apoptosis or growth rate did not yield any significant results (S4 and Table E in S1 File). We then determined the number of EBV genomes per latently infected cell of each cell line by fluorescent *in situ* hybridization FISH (Fig E in S1 File) or qPCR and correlated this number to the cell growth rate or to the BHRF1 cluster miRNA expression level (Table F and G in S1 File). We could not find any significant correlation between these parameters (Table G in S1 File). The sequence of the LMP1 open reading frame and of its promoter has previously been shown to correlate with the ability of this protein to activate the NF-kB signaling pathway [30]. Therefore, we compared the growth rate of our LCL panel with the type of LMP1 it carries (Fig F in S1 File, Table H in S1 File). This analysis showed no differences between the investigated groups in terms of growth rate.

### 5) Comparison between sLCLs isolated from patients with iEBVL after transplantation or with IM

**Fig 5:**
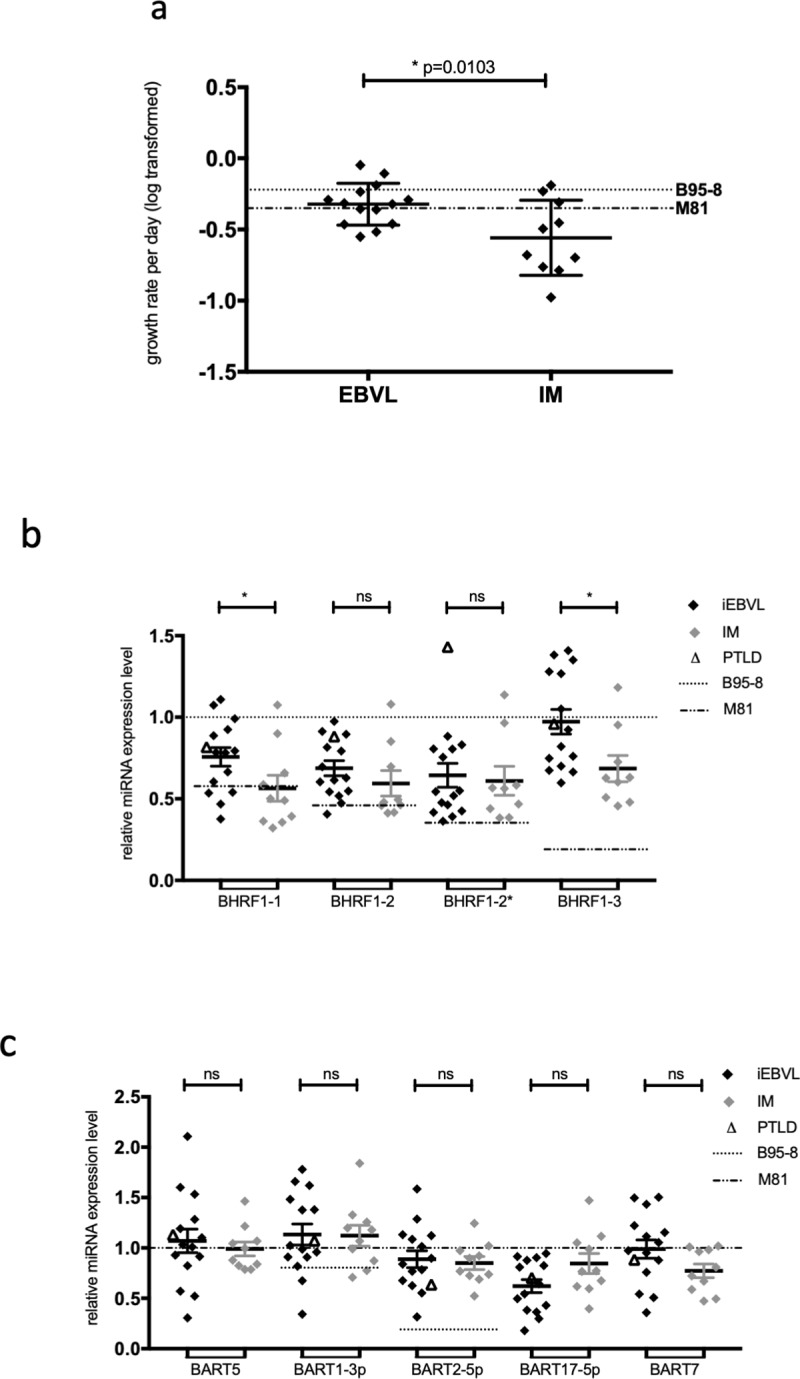
sLCLs from transplanted patients with iEBVL display higher growth rates and higher levels of miR-BHRF-1 and miR-BHRF1-3 compared to sLCL from IM patients. (a) The dot plots compare the growth rates of sLCLs isolated from transplant recipients with iEBVL or IM patients. The results of the statistical analysis are also given. (b and c) The dot plots compare the expression of the BHRF1 miRNAs and of some BART miRNAs in sLCLs isolated from transplant recipients with iEBVL or IM patients. The results of the statistical analysis are also given.

We wished to determine whether there was a difference between the growth rates of sLCLs established from transplant recipients with iEBVL or from patients with IM (Fig 5A). This analysis could identify a statistically significant higher average growth rate in the EBV-transformed cells generated from the first group of patients, according to a non-paired t-test. Similarly, comparison of the distribution pattern and of the average of BHRF1 miRNA expression levels between the two patient groups revealed that all four BHRF1 miRNAs were, on average, expressed at higher levels in iEBVL sLCLs than in IM sLCLs. These differences reached statistical significance for both miR-BHRF1-1 and -3 and were maximal for the latter miRNA (Fig 5B). We omitted the values obtained with IM-10 as they were very close to zero and could overly influence the statistical analysis. We noticed a lower expression of BART17-5p in sLCLs from transplant recipients than in IM cell lines that, however, did not reach statistical significance (Fig 5C). Altogether, there was no significant difference in BART miRNA expression between the two groups of LCLs. They expressed the BART miRNAs on average at the levels seen in LCLs infected with M81. We could not find any difference between the two groups of sLCLs in terms of apoptosis, BHRF1 or latent protein expression (Fig G in S1 File).

## Discussion

In the present study, we generated a panel of B-cell lines from immunocompetent patients with primary EBV infection or from patients with iEBVL under immunosuppression after kidney or stem cell transplantation. These cells grew out very rapidly, did not show any sign of crisis, suggesting that they closely reflect the characteristics of their *in vivo* counterparts. This gave us the opportunity to compare the growth characteristics and apoptosis status of immortalized B-cells isolated from these patients. We found that these EBV-infected B-cells grew at highly different rates, implying that they would possibly generate a reservoir of infected B-cells of variable size in immunocompetent individuals, but also tumors at highly variable speed in immunosuppressed patients. This provides a potential explanation for the previously reported variable dynamics of EBV loads, patients with rapidly increasing EBV DNA levels being more likely to receive treatment as this implied a rapid increase of the B-cell numbers [31]. Our data show that this process is probably largely due to an increased rate of cell division that is mainly the result of individual interactions between the B-cell and the virus. We also found that there was a great degree of variation in the resistance of the infected cells to apoptosis that is likely to affect tumor growth and response to therapy.

This very high heterogeneity could result from multiple influences. First, this could reflect a heterogeneous transforming capacity of different virus strains. Indeed, we previously found that viruses isolated from different tumors or from lymphoid diseases display a variable ability to transform B-cells *in vitro* and to induce tumors *in vivo* in immunocompromised animals [23, 32]. The present data would extend these data gathered with laboratory EBV strains to viruses isolated in patients. Sequencing of four highly polymorphic genes showed that the viruses are all type 1 and are closed to viral strains found in Europe and in the US. Therefore, virus heterogeneity also extends to the group of western type 1 viruses. Variable cell growth rate of our panel could also reflect characteristics of the host B-cells, e.g. variable genetic susceptibility to the viral proteins, that lead to variable speed of growth upon viral infection. Alternatively, it might reflect the presence of additional genetic abnormalities that influence the growth characteristics of the infected cell, as has been previously identified in PTLD [33].

In an attempt to identify the molecular mechanisms that contribute to these disparities, we quantified latent protein and viral miRNA expression in these B-cells [3, 6]. The expression level of the LMP1 protein strikingly varied across the isolates included in our study. However, we found no correlation between LMP1 expression and the resistance to apoptosis induced by any of the pro-apoptotic drugs (Fig C in S1 File). These results could reflect a lack of statistical power to detect subtle effects induced by LMP1 or result from the influence of other genetic elements that influence apoptosis such as the BHRF1 protein. Our investigations revealed that the EBV miRNAs are expressed within a large range in this panel of sLCLs. This confirms and extends observations made on laboratory strains that already identified variations in the miRNA expression levels in cells infected with different viruses [34]. Interestingly, the growth rate of the different sLCLs positively correlated with the levels of the BHRF1-2, -2* and -3 miRNAs, suggesting that cells grew faster when they expressed higher levels of these miRNAs. Because the miR-BHRF1-1 expression levels also positively correlated with cell growth although the degree of correlation did not reach did not reach statistical significance, it is likely that the expression level of the complete BHRF1 miRNA locus modulates B-cell proliferation across B-cell samples. This probably results from variable transcription level of the genes that encode the BHRF1 miRNAs, the BHRF1 gene itself and introns spliced from EBNA transcripts that encompass the BHRF1 gene [35, 36]. However, we found no correlation between the episome copy number and the BHRF1 miRNA expression pattern. The correlation between miRNA expression and the speed of B-cell proliferation fits with the observation that although viruses devoid of the four BHRF1 miRNAs are still transforming, they are up to 20 times less efficient than the wild type viruses in transforming B-cells [6, 7]. Our analyses also showed that cells infected with M81 expressed the BHRF1 miRNAs less strongly than those infected with B95-8 and this fits with the observation that the former cells grow less rapidly than the latter.

Several parameters distinguished sLCLs from transplant recipients with iEBVL and those from IM sLCLs. We found that the former B-cells have a higher average growth rate and express all BHRF1 miRNAs except miR-BHRF1-2* more strongly than those established from IM patients. This was particularly true for miR-BHRF1-3, the miRNA that we previously found endowed with the strongest transforming abilities [37]. Thus, it is possible that the increased cell growth seen in sLCLs from transplant recipients with iEBVL compared to IM patients resulted from an increased miR-BHRF1 expression. This might reflect the reduced immune control in transplant recipients that might facilitate the selection of more transforming strains.

The observation that infection with the same virus leads to highly variable cell division rates is surprising, considering that the mitotic process is tightly regulated in normal cells. Whether the variable rate of division can contribute to the occurrence of genetic abnormalities that have been described after EBV infection is an open question [38].

Our data also have consequences for the management of patients with an increased EBV load. The monitoring of these patients over time shows that the kinetic of the virus load greatly varies between patients, some of whom show a more rapid increase than others [11]. According to our data, this might reflect the differences in growth characteristics of the infected B-cells. We hypothesize that the emergence of tumors in transplant recipients with an increased EBV load after transplantation might be facilitated by generation of quickly dividing and apoptosis-resistant infected B-cells. Evaluating the speed at which infected B-cells divide is likely to be an important parameter in patients with iEBVL who are at risk of developing PTLD.

## Supporting information

S1 File Supplementary material. (DOCX)
