# Supplementary material for: Spontaneous lymphoblastoid cell lines from patients with Epstein-Barr virus infection show highly variable proliferation characteristics that correlate with the expression levels of viral microRNAs
Source: PLoS One. 2019 Sep 30;14(9):e0222847. doi: 10.1371/journal.pone.0222847 (PMC6768455; doi:10.1371/journal.pone.0222847)
Supplement: S1 File — (DOCX) [file pone.0222847.s001.docx]

**Supplementary material**


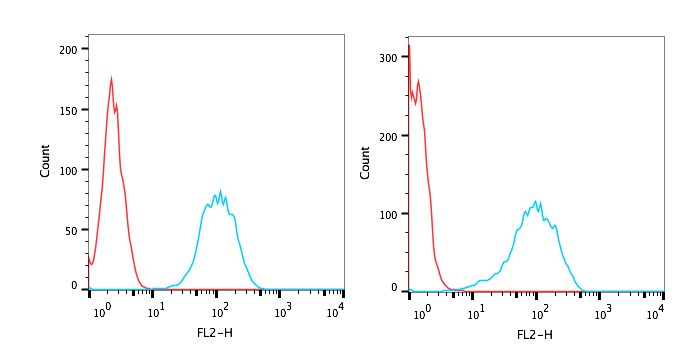


CD19

Isotype control

CD19

CD19

sLCL-9

Raji

**Figure A. EBV-transformed B cells from a transplant recipient with iEBVL express the B cell marker CD19.**

The figure shows the results of a FACS analysis with an antibody specific to CD19. The results were obtained with sLCL-9. Raji cells were used as a positive control. We included staining with an isotype control (red curve). Signals obtained with the CD19-specific antibody are shown in blue.

**Figure B. Genetic classification of EBV strains.**

The genetic tree shows the genetic distance across published EBV genomes. The top group includes viruses found in South East Asia, the bottom group consists of type 2 viruses.

**Figure C. BHRF1 and BART-cluster microRNA expression in LCLs.**

The bar graphs show the expression of each BHRF1 and BART microRNA for each LCL.

** Figure D. Correlation analyses between BHRF1 cluster miRNA expression and apoptosis.**

Apoptosis was induced in a panel of B-cells using three different drugs. The percentage of apoptotic cells in each case was determined by a TUNEL assay and correlated to the BHRF1 miRNAs BHRF1-1, -2, -2star and -3 expression levels determined by qPCR. Results of linear regression of staurosporine (a), etoposide (b) and ionomycin (c) treated cells and BHRF1-1, -2, -2star and -3 miRNAs are shown after log transformation was performed.

sLCL-7


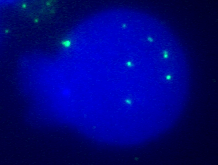


FISH

**Figure E.** **Fluorescence in situ hybridization (FISH) of EBV episomes in LCLs.**

To determine the number of EBV genome copies per cell, FISH was performed and the number of EBV genomes counted. The average copy number is given in S5 Table. The picture shows the episomes present in the sLCL-7 cell line.

**Figure F. Growth rate within the different LMP1 groups.**

Sequencing allowed the inclusion of LCLs in fours groups. Depicted are the results of a statistical analysis between these groups and the growth rate of the sLCLs and IM cell lines from which they were derived.

**Figure G. Comparison of IM and sLCLs.**

Depicted are the results of a statistical analysis between the two groups of sLCLs in terms of latent protein and BHRF1 protein expression as well as apoptosis.

Table A. Antibodies used for Western blot or FACS analyses.

| Antibody | Clone | Host species | Clonality | Dilution |
| --- | --- | --- | --- | --- |
| EBNA1 | 2B4-1 | Rat | monoclonal | 1:100 |
| EBNA2 | R3 | Rat | monoclonal | 1:200 |
| LMP1 | S12 | Mouse | monoclonal | 1:5000 |
| BHRF1 | 6A9 | Rat | monoclonal | 1:100 |
| Actin | ACTN05, C4 | Mouse | monoclonal | 1:5000 |
| CD19-PE | SJ25-C1 | Mouse | monoclonal | 1:100 |

**Table B. Sequences of primers used for quantification of EBV miRNAs by qPCR.**

| Primer | Sequence 5’-3’ |
| --- | --- |
| miR-BHRF1-2* RT | CTCAACTGGTGTCGTGGAGTCGGCAATTCAGTTGAGGCTATCTG |
| miR-BHRF1-2* fwd | ACACTCCAGCTGGGAAATTCTGTTGCAGCA |
| miR-BHRF1-2* probe | FAM – TTCAGTTGAGGCTATCTG - TAM |
| miR-BHRF1-2 RT | CTCAACTGGTGTCGTGGAGTCGGCAATTCAGTTGAGTTCAATTT |
| miR-BHRF1-2 fwd | ACACTCCAGCTGGGTATCTTTTGCGGCAGA |
| miR-BHRF1-2 probe | FAM - TTCAGTTGAGTTCAATTT - TAM |
| miR-BHRF1-1 RT | CTCAACTGGTGTCGTGGAGTCGGCAATTCAGTTGAGAACTCCGG |
| miR-BHRF1-1 fwd | ACACTCCAGCTGGGTAACCTGATCAGCCCC |
| miR-BHRF1-1 probe | FAM – TTCAGTTGAGAACTCCGG - TAM |
| miR-BHRF1-3 RT | CTCAACTGGTGTCGTGGAGTCGGCAATTCAGTTGAGGTGTGCTT |
| miR-BHRF1-3 fwd | ACACTCCAGCTGGGTAACGGGAAGTGTGTA |
| miR-BHRF1-3 probe | FAM – TTCAGTTGAGGTGTGCTT - TAM |
| miR-BART1-3p RT | CTCAACTGGTGTCGTGGAGTCGGCAATTCAGTTGAGAGACATAG |
| miR-BART1-3p fwd | ACACTCCAGCTGGGTAGCACCGCTATCCAC |
| miR-BART1-3p probe | FAM-TTCAGTTGAGAGACATAG-TAM |
| miR-BART2-5p RT | CTCAACTGGTGTCGTGGAGTCGGCAATTCAGTTGAGGCAAGGGC |
| miR-BART2-5p fwd | ACACTCCAGCTGGGTATTTTCTGCATTCGC |
| miR-BART2-5p probe | FAM-TTCAGTTGAGGCAAGGGC-TAM |
| miR-BART17-5p RT | CTCAACTGGTGTCGTGGAGTCGGCAATTCAGTTGAGCTTGTATG |
| miR-BART17-5p fwd | ACACTCCAGCTGGGUAAGAGGACGCAGGCA |
| miR-BART17-5p probe | TTCAGTTGAGCTTGTATG |
| miR-BART5 RT | CTCAACTGGTGTCGTGGAGTCGGCAATTCAGTTGAGCGATGGGC |
| miR-BART5 fwd | ACACTCCAGCTGGGCAAGGTGAATATAGCT |
| miR-BART5 probe | FAM-TTCAGTTGAGCGATGGGC-TAM |
| miR-BART4 RT | CTCAACTGGTGTCGTGGAGTCGGCAATTCAGTTGAGAGCACACC |
| miR-BART4 fwd | ACACTCCAGCTGGGGACCTGATGCTGCTGG |
| miR-BART4 probe | FAM-TTCAGTTGAGAGCACACC-TAM |
| miR-BART7 RT | CTCAACTGGTGTCGTGGAGTCGGCAATTCAGTTGAGCCCTGGAC |
| miR-BART7 fwd | ACACTCCAGCTGGGCATCATAGTCCAGTGT |
| miR-BART7 probe | FAM-TTCAGTTGAG CCCTGGAC-TAM |
| miR-universal rev | CTCAACTGGTGTCGTGGAGTCGGCA |

**Table C. Oligonucleotides, sequences and position in the reference EBV genome NC_007605.1.**

| **name** | **sequence** | **position in**  **the reference EBV genome NC_007605.1** | **position in B95-8 ORF or mRNA** | **PCR product size (bp)** | **PCR product description** |
| --- | --- | --- | --- | --- | --- |
| 3301 P-EBNA2 fwd5 | AGGGATGCCTGGACACAAGA | 36522:36541 | 307:326 in EBNA2 ORF | 1688-1694 | most of the EBNA2 C-terminus + interregion EBNA2-BHLF1 |
| 3302 P-EBNA2 rev7 | CTCTAAAAGTAACCTGTCTACTTCGCC | 38210:38184 | 2596:2570 in BHLF1 mRNA |  |  |
| 3311 P-EBNA3A fwd5 | GGCGCAAGTCCCAGAACCC | 81362:81380 | 1320:1338 in EBNA3A ORF | 2527-2614 | EBNA3A C-terminus  + EBNA3B N-terminus |
| 3312 P-EBNA3B rev2 | CCGAAAGTGACCTAGCACGAC | 83890:83870 | 748:728 in EBNA3B ORF |  |  |
| 3313 P-BZLF1 rev2 | GTAAACAATGGCACCCTCACCTG | 89791:89813 | 38:16 in BZLF2 ORF | 2474-2486 | BRLF1 C-terminus + BZLF1 gene + BZLF2 N-terminus |
| 3314 P-BRLF1 fwd | GACTATGGTCTCGTCCAAGACAGG | 92275:92252 | 621:644 in BRLF1 ORF |  |  |
| 3184 P-LMP1p fwd | CATTCCCACAGCTTGCCCC | 169283:169265 | -267:-249 before LMP1 ORF | 1568-1682 | LMP1 partial promoter  + LMP1 ORF |
| 3185 P-LMP1 rev3 | GTTAGTCATAGTAGCTTAGCTGAACTGG | 167701:167728 | +1 (after ORF):1135 in LMP1 ORF |  |  |

**Table D. Correlation between latent protein expression levels and the number of apoptotic cells after treatment with staurosporine, etoposide or ionomycin.**

|  | **Pearson r** | **95% confidence interval** | **R squared** | **P (two-tailed)** | **P value summary** |
| --- | --- | --- | --- | --- | --- |
| **EBNA1 vs. Staurosporine** | 0,3343 | -0,09030 to 0,6561 | 0,1118 | 0,1189 | ns |
| **EBNA1 vs. Etoposide** | 0,04442 | -0,3948 to 0,4671 | 0,001973 | 0,8484 | ns |
| **EBNA1 vs. Ionomycin** | 0,3784 | -0,05139 to 0,6899 | 0,1432 | 0,0825 | ns |
| **EBNA2 vs. Staurosporine** | -0,01628 | -0,4256 to 0,3986 | 0,000265 | 0,9412 | ns |
| **EBNA2 vs. Etoposide** | -0,1503 | -0,5465 to 0,3010 | 0,02258 | 0,5156 | ns |
| **EBNA2 vs. Ionomycin** | 0,3091 | -0,1293 to 0,6465 | 0,09556 | 0,1615 | ns |
| **LMP1 vs. Staurosporine** | -0,2624 | -0,6235 to 0,1909 | 0,06885 | 0,2505 | ns |
| **LMP1 vs. Etoposide** | -0,1223 | -0,5461 to 0,3515 | 0,01495 | 0,618 | ns |
| **LMP1 vs. Ionomycin** | 0,3631 | -0,09463 to 0,6941 | 0,1318 | 0,1156 | ns |
| **BHRF1  vs. Staurosporine** | 0,4031 | -0,04803 to 0,7176 | 0,1625 | 0,0781 | ns |
| **BHRF1  vs. Etoposide** | 0,1828 | -0,2960 to 0,5882 | 0,03342 | 0,4538 | ns |
| **BHRF1  vs. Ionomycin** | 0,237 | -0,2434 to 0,6241 | 0,05619 | 0,3285 | ns |

**Table E. Correlation between latent protein expression levels and growth rate.**

|  | **growth rate  vs. EBNA1** | **growth rate  vs. EBNA2** | **growth rate  vs. LMP1** | **growth rate  vs. BHRF1** |
| --- | --- | --- | --- | --- |
| Pearson r | 0,09045 | 0,07555 | 0,08198 | 0,1788 |
| 95% confidence interval | -0,3342 to 0,4846 | -0,3475 to 0,4730 | -0,3625 to 0,4961 | -0,2864 to 0,5757 |
| R squared | 0,008182 | 0,005708 | 0,00672 | 0,03196 |
| P (two-tailed) | 0,6815 | 0,7319 | 0,7239 | 0,4508 |
| P value summary | ns | ns | ns | ns |

**Table F. EBV genome copies per cell.**

|  | **EBV genome copies per cell (average)** |
| --- | --- |
| **sLCL-** |  |
| **1** | 6,00 |
| **2** | 5,50 |
| **3** | 11,70 |
| **4** | 7,46 |
| **5** | 8,80 |
| **6** | 7,20 |
| **7** | 6,25 |
| **8** | 13,18 |
| **9** | 6,15 |
| **10** | 4,82 |
| **11** | 7,30 |
| **12** | 5,60 |
| **13** | 7,81 |
| **14** | 6,47 |
| **PTLD-1** | 9,50 |
| **IM-** |  |
| **1** | 8,82 |
| **2** | 4,00 |
| **3** | 7,59 |
| **4** | 5,62 |
| **5** | 5,91 |
| **6** | 10,71 |
| **7** | 8,57 |
| **8** | 5,69 |
| **9** | 8,88 |
| **10** | 9,00 |
|  |  |
| **Average** | 7,54 |

**Table G. Correlation between EBV genome copies per cell and growth rate or BHRF1 miRNA expression levels.**

|  | **EBV genome copies per cell vs.** | | | | |
| --- | --- | --- | --- | --- | --- |
|  | **growth rate** | **BHRF1-1** | **BHRF1-2** | **BHRF1-2star** | **BHRF1-3** |
| Pearson r | 0,08287 | 0,1498 | 0,05459 | 0,2256 | 0,1099 |
| 95% confidence interval | -0,3316 to 0,4705 | -0,2699 to 0,5217 | -0,3567 to 0,4481 | -0,1956 to 0,5766 | -0,3071 to 0,4915 |
| R squared | 0,006868 | 0,02245 | 0,00298 | 0,05091 | 0,01208 |
| P (two-tailed) | 0,7003 | 0,4847 | 0,8 | 0,2891 | 0,6091 |
| P value summary | ns | ns | ns | ns | ns |

**Table H. LMP1 grouping of sLCLs and IM cell lines according to the sequence of the LMP1 gene and its promoter region.**

| **Cell line** | **LMP1 group** |
| --- | --- |
| IM-1 | B95-8 |
| IM-2 | NC |
| IM-3 | Med- |
| IM-4 | B95-8 |
| IM-5 | China 1 |
| IM-6 | China 1 |
| IM-7 | China 1 |
| IM-8 | Med- |
| IM-9 | Med- |
| IM-10 | B95-8 |
| PTLD-1 | B95-8 |
| sLCL-1 | Med- |
| sLCL-2 | Med- |
| sLCL-3 | Med- |
| sLCL-4 | Med- |
| sLCL-5 | Med- |
| sLCL-6 | B95-8 |
| sLCL-7 | China 1 |
| sLCL-8 | China 1 |
| sLCL-9 | NC |
| sLCL-10 | B95-8 |
| sLCL-11 | Med- |
| sLCL-12 | Med- |
| sLCL-13 | Med- |
| sLCL-14 | Med- |
